# Students’ performance in clinics and self-perceived Confidence in performing Endodontic procedures: A correlation study

**DOI:** 10.12669/pjms.39.1.6870

**Published:** 2023

**Authors:** Muhammad Qasim Javed, Usman Anwer Bhatti

**Affiliations:** 1 Muhammad Qasim Javed, Associate Professor, Department of Conservative Dental Sciences and Endodontics, College of Dentistry, Qassim University, Buraydah, Saudi Arabia; 2 Usman Anwer Bhatti, Associate Professor, Department of Operative Dentistry, College of Dentistry, Riphah International University, Islamabad, Pakistan

**Keywords:** Competency, Dental Education, Endodontics, Self Confidence, Undergraduate Dental Students

## Abstract

**Objective:**

The study aimed to evaluate undergraduate dental students’ self-perceived confidence in carrying out Endodontic procedures and correlating it with their performance in Endodontic clinics.

**Methods:**

The correlational study was conducted on fifth year dental students at Qassim University, Saudi Arabia. The self-perceived confidence of students was assessed by using Endodontic Self-Perceived Confidence Scale (ESCS). The students’ endodontic clinical performance scores were obtained from the department head. Data were analyzed by IBM SPSS-23.0. Descriptive statistics were recorded as percentages, frequencies, and mean. Chi-square test was used for gender-wise comparison of all items of ESCS, Student t-test was used for comparing means and Pearson correlation coefficient was utilized for exploring correlation.

**Results:**

Over all response rate was 91.8%. Students exhibited the highest confidence level for achieving rubber dam isolation (4.57±0.66) while the lowest confidence level was documented for the treatment of teeth with immature apices (2.82±0.95). The mean self-confidence score of male students on ESCS was higher than the female students (P-value=0.18). However, mean endodontic clinic score of female students was significantly higher than the male students (P-value=0.02). The insignificant correlation was noted between the students’ mean confidence scores and endodontic clinical performance scores (P-value=0.82).

**Conclusions:**

The insignificant correlation between self-perceived confidence and clinic scores raises significant caveats for utilizing the self-assessment in the aforementioned group. Hence, the students should be adequately trained in self-assessment to prepare them for lifetime learning. Additionally, clinical instructors by creating a supportive learning environment should help students to deal with their shortcomings.

## INTRODUCTION

Endodontics is an evolving discipline which demands a sound knowledge as well as skill to ensure optimal delivery of treatment. The undergraduate dental curriculum is responsible for training the future dental surgeons with the necessary knowledge and skills.^1^-^3^ Following culmination of the undergraduate education, it is expected that future dental surgeons will be able to deliver the patients, standardized quality of care by conducting proper clinical evaluation, providing proper diagnosis, formulating adequate treatment plan, and skillfully executing the endodontic treatment.^4^,^5^

The Saudi Arabian Dental Schools design their Endodontic didactic and clinical training programs in accordance with the instructions of Saudi Commission for Health Specialties.^6^ The general acceptance is that cumulative number of endodontic treatments performed by undergraduate students has an effect on their competence as it ascertains the students’ readiness to work without supervision.^2^,^7^

However, the European Society of Endodontology in its undergraduate endodontics curriculum guidelines have highlighted that the quality and consistency of undergraduate students’ clinical performance, are of greater significance than the cumulative number of endodontic treatment cases.^8^ Likewise, Gilmour and colleagues argued that the total endodontic treatments performed by students do not necessarily indicate the level of their competency.^9^

The quality of the endodontic treatment is a significant parameter for evaluating students’ proficiency.^10^ Recent researches that assessed the outcome, and quality of endodontic procedures have highlighted a high percentage of under par endodontic treatments carried out by students.^11^,^12^ This is a matter of concern as the quality of clinical training of students in any subject is reflected in their competency and self-confidence.^13^A self-confident individual is expected to accomplish a goal in a particular situation.^14^ Hay argued that self-confidence is a significant psychological construct that influences the student’s ability to demonstrate competency.^15^

Contrary to this, Woolliscroft et al. suggested that the students’ self-confidence levels (perception of their competency) might not always be directly related to their competency.^16^ However, Gabbard et al. while questioning the validity of using subjective measures of student competence recognized the importance of student perception in generating competency data.^17^

There has been no study to date that has explored the correlation of Undergraduate Dental students’ self-perceived confidence level and their performance in the Endodontic clinics. Therefore, the aim of this study was to evaluate the undergraduate dental students’ self-perceived confidence in carrying out Endodontic procedures and correlate the confidence level with their performance in Endodontic clinics.

## METHODS

The Qassim University dental school follows the competency-based curriculum. The undergraduate dental students start both didactic, and practical endodontic teaching during 3^rd^ year of the program. Students are provided with the teaching of endodontics over the last three years of their undergraduate dental course. A pre-clinical endodontic skill lab starts at the beginning of first term of 3^rd^ academic year with the comprehensive workshop on rubber dam isolation, whereas training in the clinics commences in the first term of 4^th^ academic year and continues till the time of graduation. The preclinical skills course is comprised of 60 hours of hands-on training that include the completion of root canal treatment both by manual and rotary instrumentation techniques.

During preclinical sessions, students complete endodontic treatment on seven teeth (four one rooted, and three multi-rooted extracted teeth and plastic teeth). Subsequently, in the fourth-year students are required to complete endodontic treatment on five teeth (one rooted having straight canals). In the final academic year students complete endodontic treatment on eight teeth (two one rooted having curved canals, two premolar teeth with two canals, two premolar teeth having curved canals, and two Molars). Endodontic procedures are performed under the guidance of consultants as an integral constituent of comprehensive patient care block.

### Ethical Approval:

The ethical approval was obtained from the institutional review board at the school of dentistry, Qassim University (ST/6071/2020).

The self-perceived confidence level of students while performing different stages and procedures of endodontic treatment was assessed by using a self-composed, Endodontic Self-perceived Confidence Scale (ESCS). The ESCS was devised by a team of endodontists after literature review.^2^,^3^,^18^ The ESCS was then reviewed for content relevancy and ease of understanding, by two senior endodontists and two GDPs at the College of Dentistry, Qassim University. The wording of the questions was modified according to the comments of reviewers to enhance the clarity of questions. Subsequently, a pilot study was carried out on limited sample of interns (N=14) working at the Qassim University Dental Clinics. The outcome of the pilot study was assessed by the statistician at the Qassim University and ESCS was finalized.

The sample size calculation for the current study was carried out by using a Clin Calc LLC sample size calculator.^19^ The mean self-perceived confidence score values obtained from the pilot study was used for sample size calculation. The mean value for group one (Female interns) was 95.72±1.74 and group two (Male interns) was 94.24±1.92. The male to female ratio was 1.33:1. Total of at least 44 responses from students were found to be sufficient with 0.05 type one error and 80% power of study.

The scale comprised of twenty-seven items. The students responded to each item on a five-point Likert scale as follows: Very confident=5, Confident=4, Neutral=3, Little confident=2, 1=Very little confident. The cumulative confidence score was calculated for each student by summing the student’s scores on individual items. The range of the cumulative score was between 27 and 135, with a higher score corresponding to a higher self-perceived confidence level.

The ESCS along with the consent form was sent via an email to the 49 year five undergraduate dental students, at the end of academic year. Subsequently, two consecutive weekly email reminders were given. The students were informed that participation in the study was voluntary. The responding students were included in the research and those who failed to respond were excluded. The endodontic clinic scores of the students were obtained from the department head. The 5^th^ year students in the endodontic clinics were scored out of 100 marks by using the rubrics for each of the 8 endodontic treatment cases that they were required to complete. The break-up of the 100 marks was as follow: history taking and diagnosis (15 marks), anesthesia (5 marks), working length determination (10 marks), Isolation (10 marks), access cavity preparation, canal instrumentation, and canal obturation (20 marks each). The six staff members were calibrated at the start of the academic year to ensure uniformity in the marking. The mean score for the eight cases that each student completed was calculated for all the students.

### Statistical Analysis:

Data analysis was carried out by utilizing IBM SPSS version 23. Descriptive statistics were recorded as percentages, frequencies, and mean. The level of significance was set at below 0.05 (p-value<0.05). The mean confidence score was calculated for the cohort. Chi-square test was used for gender-wise comparison of all items of ESCS and student t-test was utilized for gender-wise comparison of the cumulative mean scores on ESCS. The reliability of the ESCS was assessed by Cronbach’s alpha. The mean score for endodontic clinics was recorded for the cohort. Furthermore, a gender-wise comparison of the mean endodontic clinic score was carried out by Student t-test. Shapiro-Wilk test was used for confirming normality of mean endodontic clinic and mean confidence scores. Linearity between mean endodontic clinic and mean confidence score was checked using ANOVA. The correlation between the students’ self-perceived confidence level and endodontic clinics score was assessed by utilizing the Pearson correlation coefficient (p < 0.05).

## RESULTS

A total of 45 students responded with an overall response rate of 91.8%. However, data analysis indicated a significant outlier which was removed bringing the total sample to 44. The male to female ratio of the respondents was 1.31:1 (25 males and 19 females). The age range of the participants was between 23 and 25 years. Students’ self-perceived confidence level on the individual items of ESCS is shown in Table-I.

**Table-I:** Endodontic Self-Perceived Confidence Scale showing mean self-perceived confidence level (N=44)

Items	Mean±SD	Student’s Responses* N (%)					P-value
	
How confident are you in:		VLC(1)	LC(2)	N(3)	C (4)	VC(5)	Gender^†^
1. Performing the endodontic evaluation, history taking and diagnosis of the patients	4.05±0.71	0(0)	1(2.2)	7(15.9)	25(56.8)	11(25)	0.95
2. Achieving the anesthesia for endodontic treatment	4.22±0.89	1(2.2)	1(2.2)	4(9.09)	19(43.2)	19(43.2)	0.26
3. Preparing the endodontic access cavity.	3.98±0.82	0(0)	3(6.8)	6(13.6)	24(54.5)	11(25)	0.19
4.Achieving the rubber dam isolation	4.57±0.66	0(0)	1(2.27)	1(2.27)	14(31.8)	28(63.6)	0.20
5. Measuring the working length	4.16±0.80	0(0)	2(4.5)	5(11.4)	21(47.7)	16(36.4)	0.26
6. Performing the root canal shaping and irrigation	4.40±0.62	0(0)	0(0)	3(6.8)	20(45.4)	21(47.7)	0.55
7. Performing the root canal obturation	4.20±0.81	1(2.27)	0(0)	5(11.4)	21(47.7)	17(38.6)	0.29
8. Managing the inter-appointment flare up	3.43±0.97	0(0)	7(15.9)	19(43.1)	10(22.7)	8(18.2)	0.32
9. Radiograph taking	4.43±0.79	0(0)	1(2.2)	5(11.4)	12(27.2)	26(59.0)	0.65
10. Communicating with patients	4.43±0.70	0(0)	0(0)	5(11.4)	15(34.0)	24(54.5)	0.34
11. Restoring root canal treated teeth	4.50±0.55	0(0)	0(0)	1(2.2)	20(45.4)	23(52.2)	0.17
***Performing root canal treatment of***							
12. Maxillary Anterior Teeth	4.50±0.82	1(2.2)	0(0)	3(6.8)	12(27.2)	28(63.6)	0.86
13. Maxillary Premolars	4.30±0.70	0(0)	1(2.2)	3(6.8)	22(50)	18(40.9)	0.79
14. Maxillary Molars	3.02±0.82	0(0)	11 (25)	24 (54.5)	6 (13.6)	3 (6.8)	0.84
15. Mandibular Anterior Teeth	3.80±1.13	1 (2.2)	5 (11.4)	12 (27.2)	10(22.7)	16(36.3)	0.03
16. Mandibular Premolars	4.48±0.66	0(0)	0(0)	4 (9.1)	15(34.1)	25(56.8)	0.35
17. Mandibular Molars	3.68±0.83	0(0)	4 (9.19)	12 (27.2)	22 (50)	6 (13.6)	0.28
18.Performing Vital pulp treatments	3.95±0.80	0(0)	1 (2.2)	12 (27.2)	19(43.2)	12(27.2)	0.28
19.Performing Endodontic retreatment	3.81±0.95	1(2.2)	3(6.8)	9(20.5)	21(47.7)	10(22.7)	0.15
20.Performing Emergency cases in general	3.77±1.02	0(0)	6 (13.6)	11(25.0)	15(34.0)	12(27.2)	0.06
***Managing teeth with***							
21. Irreversible Pulpitis	4.18±0.62	0(0)	0(0)	5 (11.4)	26(59.1)	13(29.5)	0.83
22. Acute apical periodontitis and abscess	3.61±0.95	1 (2.2)	3 (6.8)	16 (36.4)	16(36.4)	8 (18.2)	0.91
23. Chronic apical lesions (Chronic apical periodontitis, abscess and cysts)	3.86±0.88	0(0)	3 (6.8)	11 (25)	19(43.2)	11 (25)	0.06
24. Endo-perio combined lesions	3.04±0.78	2 (4.5)	5 (11.4)	27 (61.4)	9 (20.4)	1 (2.2)	0.47
25. Traumatic cases	3.02±0.95	1(2.2)	13(29.5)	17(38.6)	10(22.7)	3(6.8)	0.28
26. Root resorption	2.86±1.07	5(11.4)	11(25)	15(34.1)	11(25)	2(4.5)	0.21
27. Immature apices	2.82±0.95	4(9.1)	10(22.7)	22(50)	6(13.6)	2(4.54)	0.26

Derived from

† student’s t-test,

* VLC=Very Little Confident, LC = Little Confident, N= Neutral, C = Confident, VC = Very Confident.

The students’ mean self-confidence score was found to be highest for achieving the rubber dam isolation (4.57±0.66), whereas the low confidence level was documented for the treatment of teeth with immature apices (2.82±0.95) and root resorption (2.86±1.07). The item-wise difference in the mean confidence score of male and female participants was found to be statistically significant only for treatment of mandibular anterior teeth (Item 15).

The ESCS was found to be reliable with the Cronbach Alpha value of 0.86. The mean confidence score for the cohort on ESCS was found to be 105.10±10.45 (Table-II). Normal distribution of mean confidence score was confirmed by Shapiro-Wilk Test (p-value=0.82). The mean confidence score of male students was higher than the female students on ESCE but the difference was found to be statistically insignificant (p-value=0.18) (Table-III).

**Table-II:** Mean overall endodontic clinics score and self-perceived confidence score on ESCS (N=44).

Variable	Mean ± SD	Minimum	Maximum
Endodontics Clinics Score>	94.90±2.01	90	99
Self-perceived confidence Score	105.10±10.45	80	127

**Table-III:** Gender-wise comparison of endodontic clinics score and self-perceived confidence score on ESCS (N=44).

Variables	Gender	N	Mean ± SD	Minimum	Maximum	P-value ^†^
Endodontics Clinics Score	Male	25	94.06±2.38	90	99	0.02*
	Female	19	95.68±1.67	92	98	
Self-Perceived Confidence Score	Male	25	106.96±9.60	90	127	0.18
	Female	19	102.63±11.26	80	125	

† Student t-test,

* Level of significance< 0.05

The mean score obtained by the cohort in the endodontic clinics was 94.90±2.01 (Table-II). Normal distribution of mean endodontic clinics score was confirmed by Shapiro-Wilk Test (p-value=0.17). The mean endodontic clinics’ score of female students was found to be significantly higher than the male students (p-value=0.02) (Table-III). Pearson correlation showed a very weak negative correlation between the Endodontic clinic score and confidence score of the cohort on ESCS but this correlation was found to be statistically insignificant (p-value=0.82) (Table-IV).

**Table-IV:** Correlation of Self-perceived confidence level and Endodontic clinics score.

Variables	Correlation Coefficient (r)	*P-value
Self confidence level and Endodontic clinics score	-0.03	0.82

* Level of significance< 0.05.

## DISCUSSION

The assessment of competence is an important step for validating the quality of graduating students. The role of the competency-based curriculum (CBC) is to identify the key components of dental practice while laying out a well-defined course with a series of learning outcomes that will enable the learners to graduate as a qualified beginner. Moreover, recent study suggests that CBC may improve the metacognitive ability and educational performance of students.^20^ The enhanced metacognitive capability of students enables them to do self-assessment that is pivotal for a hands-on clinical learning.^21^ Accordingly, it is crucial for learners to understand their cognitive processes and develop optimal skills for carrying out self-evaluation of confidence level and competence.

The self-confidence affects the individual’s capacity to think optimistically, persevere in difficult times and eventually, to accomplish the tasks.^14^ Whilst academicians assess students’ work preparedness through examinations and evaluations, it is equally important to assess the students’ self-perceived confidence (perception of competency) for performing a particular task. However, one’s judgment of self-confidence is a complicated task and students’ self-perceived confidence levels might not always be directly related to their competency.^16^ Therefore, the current study has examined the self-perceived confidence of students and correlated it with performance scores in endodontic clinics. For the current study, all students in the final year of graduation were invited. The response rate was adequate^22^, and the internal consistency of Endodontic Self-Perceived Confidence Scale (Cronbach’s alpha=0.86) was found to be satisfactory.^23^

Considering, the self-perceived confidence for the individual stages of endodontic treatment the highest level of confidence was reported by the students for the rubber dam application. Overall, 95.4% of year five undergraduate dental students were confident/very confident while isolating the teeth with rubber dam that was lower than the 100% student confidence reported in New Zealand for the year five students.^24^ The finding was in accordance with the results of the study conducted by Almohaimede where 96.6% of the year five students were confident/very confident.^25^ Contrary to this, Alrahabi M et al. reported a lower level of confidence in 5^th^ year students for rubber dam isolation (69.5% confident/very confident).^26^

The high level of confidence in present research might be the outcome of the comprehensive hands-on training on the rubber dam isolation before the commencement of clinics. The low confidence level in the present study was documented for treatment of teeth with immature apices (18.14% of students confident/very confident), and root resorption (29.5% of students confident/very confident). Likewise, 5^th^ year students documented lower confidence level in New Zealand for managing the teeth with root resorption (7.8% confident/very confident).^24^ The low confidence level in aforementioned cases should not be a matter of concern as they are not included in the core competencies that dental students are expected to achieve before graduation.^5^However, the graduating dentist should achieve competence for recognizing and taking adequate action for the management of cases that require complicated non-surgical endodontic treatment and surgical treatment.^5^

The gender-wise analysis of the mean confidence score on ESCS showed that the confidence level of male students was higher than the female students. Comparable gender disparity was documented in the earlier research work, where higher level of self-perceived confidence was exhibited by the male students while carrying out relatively complex endodontic procedures.^9^,^27^ Similar gender-based differences were reported in a cohort of medical students, where female students constantly expressed lower level of confidence in competencies as compared to male counterparts.^28^ On the other hand, the gender-wise analysis of mean endodontic clinic score highlighted that the female students scored significantly higher than their male counterparts. This incongruity between the female students’ self-confidence and undergraduate endodontic clinic score can be a result of the imposter phenomenon (IP) that was first reported in women.^29^ Impostorism is a psychological phenomenon in which a person is skeptical of his/her accomplishments and abilities, despite proof of competence and success.^30^

The findings of a recent study conducted at the Harvard Dental and Medical school have highlighted the female gender as the most significant predictor of the intense imposter phenomenon.^31^ Likewise, the study on dental students and house officers in Pakistan noted that female gender has slightly more predisposition to IP than males.^32^ Considering this, the aforementioned confidence-score disparity can be attributed to the phenomenon of impostorism. Alternatively, the finding of the study can be explained by the theory put forward by Bandura and Locke where they suggested that little self-doubt (self-confidence reduction) might result in an increased effort by an individual which may ultimately improve performance.^33^ The results of the study by Woodman and colleagues have supported the theory of Bandura and Locke.^34^

The insignificant correlation was noted between the mean Endodontic clinic score and mean confidence score of the cohort on ESCS. The finding raises concerns regarding the capability of dental students to accurately evaluate their competence for a particular skill and demonstrates the requirement of their better training in self-evaluation in order to prepare them for lifelong learning. Similar results were reported by Liaw et al. where they noted an insignificant correlation between the clinical performance and self-confidence of 3^rd^ year nursing students.^35^ The outcome is also consistent with the findings of previous studies on the junior medical officers and medical students, implying that self-assessment of confidence might not be the valid predictor of students’ clinical performance.^36^,^37^ Thus far, there is no comparable data which has explored the correlation between undergraduate dental students’ self-confidence level and endodontics clinic score. Given the limited research in this area, the evidence to understand the correlation between self-perceived confidence and clinical performance is limited that warrants additional investigations.

### Limitations of study:

Firstly, it is quantitative single centered study. Considering this the findings may not be generalized to all the dental students. Secondly, the study was conducted on fifth year students only and the sample size was limited.

### Strengths of study:

This is the first study which has explored the correlation between undergraduate dental students’ self-confidence level and endodontics clinic score. Despite the aforementioned limitations, the present research can provide the foundation for future studies.

## CONCLUSIONS

The study indicated an insignificant correlation between self-perceived confidence and scores achieved by undergraduate dental students in endodontic clinic. The finding raises significant caveats for utilizing the self-assessment in the aforementioned group. The study results will be used for proposing changes in the dental curriculum to adequately train the students in self-assessment to prepare them for lifetime learning through reflective practice. Reflection will help the learners to recognize their shortcomings and rectify them. Additionally, the clinical instructors are recommended to help the students to deal with their shortcomings by using positive thinking and fighting negative beliefs (impostorism). This can be achieved by focusing on building students’ self-confidence through creating a supportive learning environment and incorporating new teaching methodologies and training approaches to enhance endodontic education experience of students.

### Future Recommendations:

It is recommended that multicenter mixed method studies should be conducted to explore in depth, how self-perceived confidence is associated with performance indicators, and the phenomenon behind this relationship. Moreover, inclusion of all the students working in clinics can provide an enhanced understanding of self-perceived confidence and clinical performance correlation.

### Authors Contribution:

**MQJ** conceived, designed, did the data collection, statistical analysis, write up & final approval of manuscript.

**UAB** write up, editing, and review of manuscript.
